# Service delivery approaches related to hearing aids in low- and middle-income countries or resource-limited settings: A systematic scoping review

**DOI:** 10.1371/journal.pgph.0002823

**Published:** 2024-01-24

**Authors:** Lauren K. Dillard, Carolina M. Der, Ariane Laplante-Lévesque, De Wet Swanepoel, Peter R. Thorne, Bradley McPherson, Victor de Andrade, John Newall, Hubert D. Ramos, Annette Kaspar, Carrie L. Nieman, Jackie L. Clark, Shelly Chadha

**Affiliations:** 1 Department of Otolaryngology- Head & Neck Surgery, Medical University of South Carolina, Charleston, South Carolina, United States of America; 2 Department of Noncommunicable Diseases, World Health Organization, Geneva, Switzerland; 3 Health Workforce and Service Delivery Unit, Division of Country Health Policies and Systems, World Health Organization Regional Office for Europe, Copenhagen, Denmark; 4 Department of Behavioral Sciences and Learning, Linköping University, Linköping, Sweden; 5 Department of Speech-Language Pathology and Audiology, University of Pretoria, Pretoria, South Africa; 6 Department of Otolaryngology—Head & Neck Surgery, University of Colorado School of Medicine, Aurora, Colorado, United States of America; 7 Section of Audiology and Eisdell Moore Centre, University of Auckland, Auckland, New Zealand; 8 Centre for Hearing Research, School of Health & Rehabilitation Sciences, University of Queensland, Brisbane, Australia; 9 Department of Speech Pathology and Audiology School of Human and Community Development, University of the Witwatersrand, Johannesburg, South Africa; 10 Department of Linguistics, Macquarie University, Sydney, Australia; 11 Master in Clinical Audiology Program, Faculty of Medicine and Surgery, University of Santo Tomas, Manila, Philippines; 12 ENT Clinic, Tupua Tamasese Meaole Hospital, Apia, Samoa; 13 Department of Otolaryngology-Head & Neck Surgery, Johns Hopkins School of Medicine, Baltimore, Maryland, United States of America; 14 University of Texas at Dallas–AuD Program, Dallas, Texas, United States of America; PLOS: Public Library of Science, UNITED STATES

## Abstract

Hearing loss is an important global public health issue which can be alleviated through treatment with hearing aids. However, most people who would benefit from hearing aids do not receive them, in part due to challenges in accessing hearing aids and related services, which are most salient in low- and middle-income countries (LMIC) and other resource-limited settings. Innovative approaches for hearing aid service delivery can overcome many of the challenges related to access, including that of limited human resources trained to provide ear and hearing care. The purpose of this systematic scoping review is to synthesize evidence on service delivery approaches for hearing aid provision in LMIC and resource-limited settings. We searched 3 databases (PubMed, Scopus, Ovid MEDLINE) for peer-reviewed articles from 2000 to 2022 that focused on service delivery approaches related to hearing aids in LMIC or resource-limited settings. Fifteen peer-reviewed articles were included, which described hospital-based (3 studies), large-scale donation program (1 studies), community-based (7 studies), and remote (telehealth; 4 studies) service delivery approaches. Key findings are that hearing aid services can be successfully delivered in hospital- and community-based settings, and remotely, and that both qualified hearing care providers and trained non-specialists can provide quality hearing aid services. Service delivery approaches focused on community-based and remote care, and task sharing among qualified hearing care providers and trained non-specialists can likely improve access to hearing aids worldwide, thereby reducing the burden of untreated hearing loss.

## Introduction

Hearing loss is an important global health issue that disproportionately affects individuals in low- and middle-income countries (LMIC) [1]. While there are several rehabilitation approaches for individuals with hearing loss, the use of hearing aids is an effective strategy that could successfully mitigate the negative consequences of untreated hearing loss for most individuals worldwide [2]. However, the World Health Organization (WHO) estimates only 9% and 15% of individuals residing in low- and middle-income countries, respectively, who would benefit from hearing aids actually receive them [2]. In part, this considerable unmet need is due to challenges in access and affordability of hearing aids and related services. Innovative approaches for hearing aid service delivery can overcome some of the constraints that limit use of hearing aids in LMIC or resource-limited settings, including those related to limited access, prohibitive cost, and inadequate human resources.

The lack of an appropriately trained workforce and the unavailability of trained providers worldwide limits access to hearing health care services. There are immense gaps in the availability of hearing health care professionals, including ear-nose-throat (ENT) specialists and audiologists, worldwide. Ninety-five percent and 65% of high-income countries have more than 10 ENT specialists or audiologists, respectively, per 1 million population. In contrast, 78% and 93% of low-income countries have less than 1 ENT specialist or audiologist, respectively, per 1 million population [2, 3]. The cost of hearing aids is a barrier and varies globally. In the United States, the price of a single hearing aid can range from 500 to 3000 USD [4]. While lower-cost options are available in some areas of the world, the cost of hearing aids and maintenance (e.g., batteries, repairs) remains unaffordable for many [5, 6].

There is a pressing need to promote the treatment of hearing loss worldwide given that untreated hearing loss has serious negative consequences on individuals across the lifespan. In children, untreated hearing loss may negatively impact oral language development and communication and has been associated with lower literacy and educational attainment [7]. In adults, untreated hearing loss has been associated with poorer quality of life and psychosocial well-being and employability [8], as well as serious health conditions such as cognitive decline and dementia [9–12]. Hearing loss also has societal consequences, as the global cost of untreated hearing loss is estimated at nearly one trillion US dollars annually [2, 13].

Hearing aids are a cost-effective approach to mitigate the burden of hearing loss on individuals and society [14–16]. Optimizing sustainable service delivery approaches related to hearing aid provision could improve access to hearing aids, the importance of which is recognized and demonstrated by changes service delivery in the United States that allow for the direct purchase of over-the-counter hearing aids, which will likely influence international changes to hearing aid service delivery [17]. Optimizing service delivery approaches for hearing aids and supporting innovations to advance low-cost hearing technology is particularly important in LMIC and resource-limited settings, where access to hearing health care, including to hearing aids, is especially poor [2]. Services related to hearing aids could be delivered in a variety of contexts including hospital or clinical settings, in community settings, or remotely (i.e., satellite care via telehealth) or using a combination of several of these methods [18].

A few systematic reviews on service delivery approaches for hearing health care have recently been published [19, 20]. A systematic scoping review focused on community-based hearing health care concluded that the limited published evidence likely supports the feasibility of providing community-based rehabilitation services [19]. Another systematic review focused on telehealth for hearing rehabilitation concluded that it is likely feasible to provide hearing aid fitting and follow-up services via telehealth [20]. Taken together, those reviews suggest community-based and telehealth approaches are likely feasible for hearing aid provision, but both highlight the need for additional high-quality research to support the implementation of those methods [19, 20]. Importantly, those reviews are relatively broad in scope and provide limited detail on hearing aid provision specifically.

A better understanding of hearing aid service delivery approaches could inform guidance and decision-making related to service delivery standards for hearing aid provision in LMIC or resource-limited settings. Therefore, the purpose of this systematic scoping review was to synthesize evidence on service delivery approaches for hearing aid provision in LMIC and resource-limited settings.

## Materials and methods

This scoping review was conducted according to the Preferred Reporting Items for Systematic Reviews and Meta-Analyses guidelines for scoping reviews (PRISMA-ScR) [21]. The protocol was pre-registered with Open Science Framework (registration: https://doi.org/10.17605/OSF.IO/PY3NA). A critical appraisal of the quality or risk of bias of individual sources of evidence was not conducted given the nature of this scoping review.

### Eligibility criteria

Peer-reviewed manuscripts or grey literature published in English, Spanish, or French that adopted observational, mixed-methods, trials, or case study designs, and that presented results from original research were eligible for inclusion. Studies must have been published between January 2000 and December 2022; these dates were chosen to capture relatively recent publication dates. Studies were included if they described service delivery approaches related to hearing aid provision in LMIC or resource-limited settings. LMICs were defined by country income level, as determined by the World Bank in terms of gross national income (GNI) per capita as follows, low-income: ≤ $1,135, lower-middle income: $1,136 to $4,465, upper-middle income: $4,466 to $13,845, and high-income: > $13,846 [22]. Studies conducted in resource-limited settings were determined by the authors’ description of the study setting. Importantly, some resource-limited settings were located in high-income or upper-middle income countries, as defined above [22].

### Information sources and search

The electronic databases PubMed, Scopus and Ovid MEDLINE were searched in June 2022 using a combination of MeSH terms and key words in English. Pilot searches confirmed the sensitivity and specificity of search terms. Search strings are in S1 Text. Grey literature sources including newsletters, reports, or proceedings were searched using similar keywords in attempts to identify high-quality, relevant evidence that was not available in published, peer-reviewed articles. An updated search was conducted in December 2022 to identify any articles published since June 2022. Search terms were translated into French or Spanish, and were used for hand searches in Google Scholar. The articles identified were assessed for eligibility by a single author who is a native Spanish speaker and proficient French speaker. Reference lists and citations of included studies (from Google Scholar) were searched to identify additional relevant articles.

### Selection of sources of evidence

Titles and abstracts of peer-reviewed manuscripts were screened by a single reviewer (LKD). Full texts were screened by two reviewers (LKD, CMD) and any differences were reconciled via discussion of manuscripts that was focused on whether manuscripts fit the inclusion criteria. Titles and abstracts, and full texts in French or Spanish were screened by a single reviewer (CMD).

### Data charting process and data items

Data collection tables were developed and were piloted to extract data from several articles. A single reviewer extracted study data (LKD). The extracted data were verified for correctness and comprehensiveness by a second reviewer (CMD). Data collection tables included details related to: a) meta study information (e.g., author, year, journal), b) study and sample characteristics (e.g., study design, participant age, location and study setting), c) characteristics and details for all steps of hearing aid related service provision including case identification, evaluation, hearing aid fitting, follow up, and counselling, d) details on who provided services, classified as qualified hearing care providers (e.g., audiologists, ENT physicians, audiology technicians) or trained non-specialists (e.g., community health workers [CHW]), and e) outcomes. The charted data were synthesized in the tables presented in the results section.

## Results

**Fig 1:**
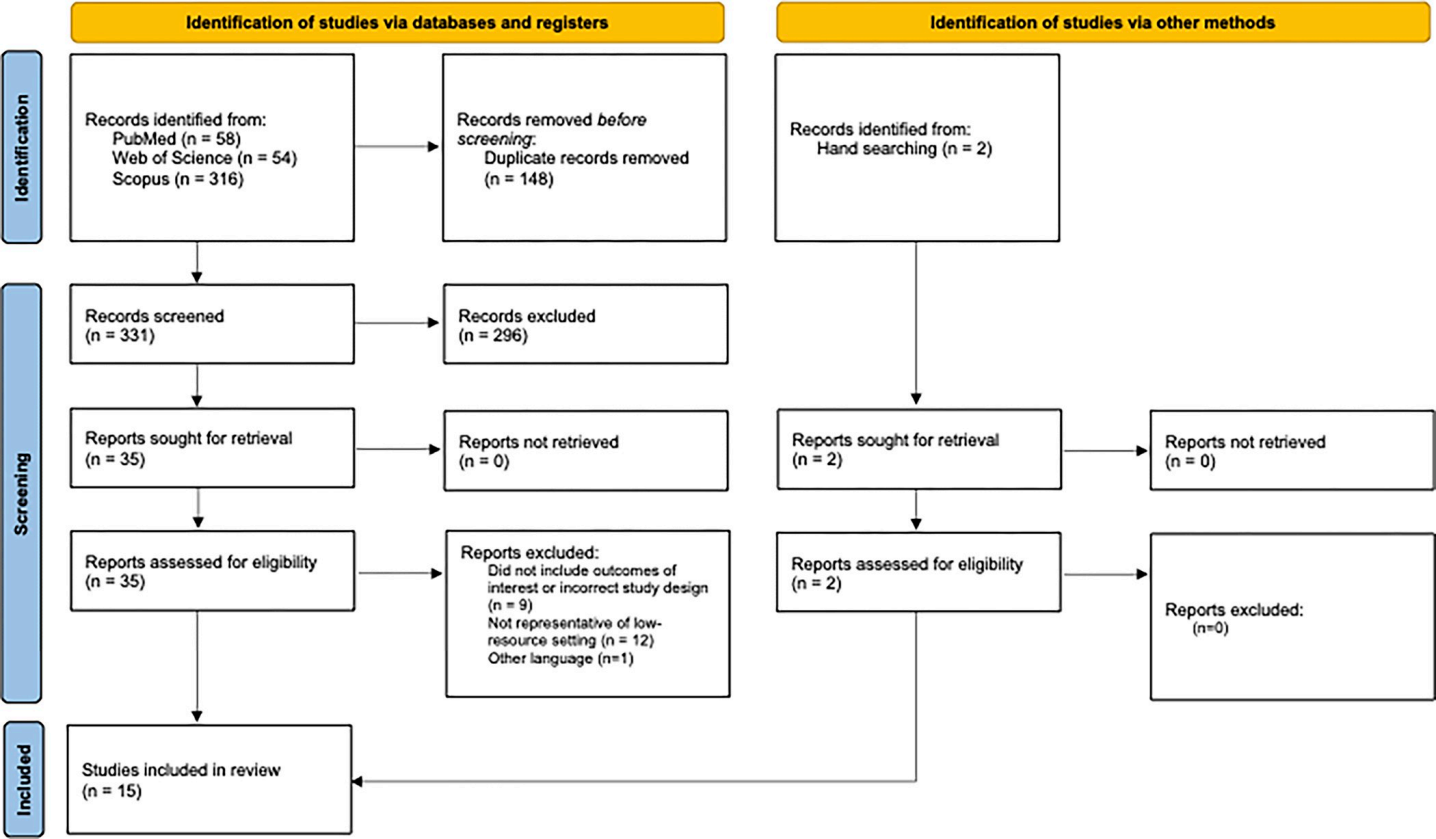
Study selection process.

A total of 331 non-duplicate citations were identified. After the final review, 15 peer-reviewed studies published in English were included, 13 of which were identified by the systematic search, and 2 of which were identified by hand searching. There were no peer-reviewed manuscripts published in Spanish or French, nor were there grey literature sources, that met the inclusion criteria. The study selection process is shown in Fig 1.

Studies were from 9 countries, corresponding to representation from African (n  =  4 studies), American (n  =  6 studies), South-East Asian (n  = 3 studies) and Western Pacific (n  = 2 studies) regions. One study was conducted in a low-income country, 4 in lower-middle income countries, 5 in upper-middle income countries, and 5 in resource-limited settings of high-income countries.

As presented below, studies were categorized by the following themes of service delivery approaches for hearing aid provision, 1) hospital-based (central) service delivery and large-scale donation programs, 2) community-based service delivery, and 3) telehealth.

### Hospital-based (central) service delivery and large-scale donation programs

Three studies focused on hospital-based (central) hearing aid provision and one study had a slightly different theme, as it focused on a large-scale hearing aid donation program supported by philanthropic organizations. Relevant details on i) service delivery and ii) study design and outcomes are in Table 1 and S1 Table, respectively. Two studies described development and sustainability of audiology departments embedded into tertiary or secondary hospitals in the Dominican Republic and Malawi [23, 24]. One study, conducted in a public hospital in South Africa, described hearing aid outcomes and barriers to hearing health care [25]. Lastly, one study described outcomes of two large-scale hearing aid donation programs in the Philippines [26]. Across these four studies, hearing aids were provided to both adults and children.

**Table 1:** Details on service provision for studies conducted in hospital-based settings or large-scale donation programs.

First Author (yr) Country (Income level)	Service provider	Program development & training	Case identification & hearing assessment	Hearing aid details	Hearing aid fitting	Hearing aid follow up and counseling
Carkeet (2014) [23] Dominican Republic (Upper-middle)	Qualified hearing care providers: Audiology technicians, audiologists, or students in 2^nd^ year of training	Developed through international collaboration. Training included 2-year audiology technician or 4-year degree in audiology.	Some screening occurs in the community. Patients are assessed in clinic, which provides a complete audiometric assessment battery.	Brand and model of hearing aids has changed over time (depending on reliability, servicing, pricing, supply). Ear mould laboratory is on-site.	Fitted to NAL algorithm. Speech testing conducted in Spanish. Real ear measures validate fitting.	Two follow-up appointments, focus on adjustments and counseling.
Parmar (2021) [24] Malawi (Low)	Qualified hearing care providers: Audiologists, audiology officers^1^; trained non-specialist: ear mould technician	Developed through international collaboration. Various levels of specialist training and non-specialist training were introduced.	Some hearing assessment occurs in community. Patients are assessed in clinic with a complete audiometric assessment battery.	Uses donated hearing aids from Hearing Aid Refurbishment Programme. Custom ear moulds made on site.	---	---
Sooful (2009) [25] South Africa (Upper-Middle)	Qualified hearing care providers: Audiologists	---	---	Government-fitted hearing aids from public hospitals.	---	---
Newall (2019) [26] Philippines (Lower-middle)	Qualified hearing care providers: Audiologists or hearing aid dispensers who were volunteers of philanthropic organization	---	Large screening camps in community.	Donated analog hearing aids which ranged from low to high power. Custom earmoulds not provided.	Fitted with lowest power hearing aids, and volume was increased until comfortable. Next powerful device was fitted as needed.	Details on follow up from philanthropic organization not provided.^2^

^1^ Nurses or clinical officers who received diploma qualifications in audiology or hearing aid acoustics

^2^ Researchers (not affiliated with philanthropic organization) followed up individuals who received hearing aids

Abbreviations: NAL: National Acoustics Laboratories

#### Service providers

In hospital settings, services were provided by qualified hearing care providers and trained non-specialists, who often worked together [23–25]. Similarly, in the study focusing on donation programs, hearing aids were fitted by qualified hearing care providers who were volunteers of an international philanthropic organization [26].

#### Program development and training

Two studies described how audiology departments were embedded into existing hospitals, and methods for training qualified hearing care providers in new audiology programs [23, 24]. The development of an audiology clinic in the Dominican Republic was facilitated by a short-term qualified hearing care provider volunteer, who aided with startup and training [23]. New programs to train qualified hearing care providers included: i) a 3-month, in-house audiometry training, ii) a 2-year audiology technician training program, and iii) a 4-year degree in audiology [23]. The development of an audiology department in Malawi was led by a qualified hearing care provider and was later supported by task sharing among the ENT physician and trained non-specialists. Efforts were supported by collaboration with several stakeholders, including an international non-governmental organization partner, which funded Masters-level training in audiology at the University of Manchester for the first Malawian audiologists. Currently, a Malawian audiologist leads the departmental operations, and is supported by other qualified hearing care providers and trained non-specialists [24].

#### Case identification and hearing assessment

Three studies described case identification and hearing assessment [23, 24, 26]. In hospital settings, most patients were self-referred, although some were identified by community-based screening or assessment [23, 24]. Hospital-based hearing assessment included otoscopy, tympanometry, pure-tone audiometry, and otoacoustic emissions [23, 24]. In the study on large-scale donation programs, case identification and hearing assessment occurred at screening camps in the community, during which participants underwent screening pure-tone audiometry [26].

#### Hearing aid details

Three studies included information on hearing aids and earmolds [23, 24, 26]. One study described changes to selection and procurement of hearing aids in the Dominican Republic during the development of the audiology department, highlighting that selection and procurement depended on reliability of the hearing aids, servicing, pricing, and supply [23]. In addition to new hearing aids, donated and reconditioned behind-the-ear hearing aids were available [23]. In the audiology department in Malawi, hearing aids were donated through a Hearing Aid Recycling Program, sponsored by an NGO partner, which also supports a small audiology laboratory that refurbishes donated hearing aids [24].

In two studies, earmolds were made on site [23, 24]. Earmolds were made following WHO guidance [23, 27], or by using locally available, low-cost dental alginate for ear impressions, and dental acrylic to make earmolds, to overcome resource limitations [24].

In the study focused on large-scale donation programs, donated hearing aids were analog, ranged from low to high power, and had volume control. Although hearing care providers took earmold impressions, custom earmolds were not distributed, but rather, standard sized, generic earmolds were selected based on patients’ ear impressions [26].

#### Hearing aid fitting

Two studies provided details on hearing aid fitting [23, 26]. In a hospital-based setting, hearing aids were fitted under standard procedures, including hearing aid verification and validation (i.e., real ear measures; translated to the local language [Spanish]) [23]. In large-scale hearing aid donation programs, patients were fitted with the lowest power hearing aid available, the volume was increased until the patient reported that the volume was comfortable. If the patient did not report a comfortable volume with that device, the next most powerful device was fitted, and the process repeated [26].

#### Hearing aid follow up and counseling

One study described standard-of-care hearing aid follow-up processes, in which patients attended 2 follow-up appointments focused on hearing aid adjustments and counseling. Qualified hearing care providers were trained to repair hearing aids, and low-cost batteries were available [23].

#### Outcomes

The outcomes reported in hospital-based studies and large-scale donation programs related to successful hearing aid use and the quality of hearing aid fitting. Two studies evaluated outcomes of hearing aids provided at a public hospital in South Africa [25], and through large-scale hearing aid donation programs in the Philippines [26]. In the public hospital, authors described that generally, hearing aids were poorly maintained and needed repair or replacement, and that a low proportion (12%) of patients used hearing aids daily. Barriers to hearing aid use and maintenance included access (e.g., transportation), language barriers, financial constraints (e.g., costs related to batteries, repairs and travel to hospital), and cosmetic concerns [25]. In the other study, researchers followed up with patients who received hearing aids from large-scale donation programs and reported a large proportion of individuals had difficulties managing their hearing aids and obtaining batteries. Furthermore, few patients were appropriately fitted to prescribed target thresholds, and many experienced hearing aid discomfort (e.g., feedback, listening in noise, from earmolds) [26].

### Community-based service delivery

Seven studies focused on community-based hearing aid delivery (Table 2, S2 Table). Five studies were randomized trials: three compared hearing aid outcomes of community-based care provided by trained non-specialists, with clinic-based care provided by qualified hearing care providers [28–30], and two evaluated feasibility of and hearing aid-related outcomes of a community-based intervention by comparing intervention and waitlist control groups [31, 32]. The two additional studies evaluated feasibility of community-based approaches, where trained non-specialists provided services, without comparison to a control group [33, 34].

**Table 2:** Details on service provision for studies conducted in community-based settings.

First Author (yr) Country (Income level)	Service provider	Program development & training	Case identification & hearing assessment	Hearing aid details	Hearing aid fitting	Hearing aid follow up and counseling
Borg (2018) [28] Bangladesh (Lower-middle)	Center-based: qualified hearing care provider Community-based: trained non-specialist (CHW)	Trained non-specialist participated in 2-day training in community-based hearing provision.	Eligibility screening conducted by qualified hearing care provider at community sites. Center-based: Hearing assessment at clinical site. Community-based: Hearing assessment in participant home.	Pocket model analog hearing aids Siemens Amiga 176 AO or 178 PP-AO	Center-based: Hearing aids coupled to custom earmoulds fitted at clinical site. Community-based: Hearing aids coupled to domes fitted in participants’ home.	---
Ekman (2017) [29] Bangladesh (Lower-middle)	Same as Borg (2018)	Same as Borg (2018)	Same as Borg (2018)	Same as Borg (2018)	Same as Borg (2018)	---
Emerson (2013) [33] India (Lower-middle)	Trained non-specialist (CHW)	Trained non-specialists participated in 6-week training on basic hearing health care.	Eligibility screening conducted in partnership with local and governmental NGOs. Few details on hearing assessment.	Behind-the-ear Siemens Phoenix 213 semi-digital	Fitted to NAL algorithm. Trained non-specialist instructed patients on hearing aid maintenance and simple adjustments.	Follow-up 6 months after fitting to obtain outcome data.
Frisby (2022) [34] South Africa (Upper-middle)	Trained non-specialist (CHW)	Trained non-specialist participated in 3-day training.	Community-based screening conducted using hearing aids that were fitted.	Behind-the-ear Lexie Lumen hearing aids	Hearing aids fitted by trained non-specialist via Bluetooth and smartphone application. Domes coupled to hearing aids.	Trained non-specialist contacted participants by telephone on days 8, 20 and 43 post-fitting. Participants received information via text for 6 weeks post-fitting. Home-based follow up 45 days and 6 months post-fitting.
Nieman (2017) [31] USA (High)	Trained interventionist	Developed and implemented protocol for use by trained non-specialist CHW.	Eligibility determined using automated protocol on tablet-based audiometer and targeted otologic review	Choice between monaural ear-level device or pocket talker: Sound World Solutions CS-50 or Wiliams Sound Pockettalker Ultra Duo	Intervention included device fitting and orientation, education, and counseling.	Telephone follow up within 5 days of intervention. Follow up 3 months after intervention to obtain outcome data.
Nieman (2022) [32] USA (High)	Trained non-specialist (CHW)	Older adult non-specialist CHW were leaders in their communities. Training included 8 weekly interactive sessions and a summative evaluation.	Similar to Nieman (2017)	Choice between monaural ear-level device or body-word amplifier with wired headset: Sound World Solutions Sidekick or Sonic Technology SuperEar SE9000	Similar to Nieman (2017)	Telephone follow up within 1 week of intervention. Follow-up 3 months after intervention to obtain outcome data.
Coco (2022) [30] USA (High)	Control group: Qualified hearing care provider Intervention group: Trained non-specialist facilitator CHW	Trained non-specialist telehealth facilitators underwent 4-day multi-level training on age-related hearing loss, technology, and tasks to complete the protocol.	Audiologists conducted assessment using portable computer-based audiometer	Mini behind-the-ear (BTE) hearing aids coupled to dome.^1^	Fitted via telehealth. Trained non-specialist facilitator was with participant and qualified hearing care provider was in a clinic.	Follow-up 3 weeks after hearing aid fitting focused on counselling, hearing aid adjustments, review of goals and expectation management.

^1^Two participants required slightly different fittings

Abbreviations: CHW: Community Health worker; NAL: National Acoustics Laboratories; NGO: Non-governmental organization

Four studies were conducted in LMIC [24, 28, 29, 33] and 3 were conducted in the United States in resource-limited settings, which were primarily low-income communities in rural [30] or urban settings [31–32]. Five studies evaluated samples of adults [30–34] and two evaluated samples of children [28, 29].

#### Service providers

In six studies, care was provided by trained non-specialists [28–30, 32–34], and in one, the protocol was developed for a trained non-specialist CHW, but in the context of the study, care was provided by an interventionist with training as a researcher and qualified hearing care provider [31]. Two studies highlighted trained non-specialist providers were native speakers of the language spoken by the served community [30, 34]. In one study, trained non-specialist providers were recognized leaders in their communities [32]. Three studies specified that trained non-specialist providers were supervised by qualified hearing care providers [30, 32, 34].

#### Program development and training

In two studies, trained non-specialists had past experience or training in disability or rehabilitation, and additional training was based on the WHO Primary Ear and Hearing Care Training Package [28, 29, 35–37].

Study-specific training protocols were also deployed. For example, non-specialists with relevant background (e.g., science, hearing health) participated in additional training courses that ranged from 4 days to 6 weeks, and that focused on study protocols, pure-tone audiometry, hearing aid fitting, earmolds, minor hearing aid repairs and maintenance, and counseling [33, 34]. One study focused on telehealth, and non-specialists with experience in community-based hearing health participated in a 4-day training on hearing loss and telehealth, which included in-person clinical observation, and assessments of knowledge and hands-on skills [30]. In another study, a hearing intervention was delivered by non-specialists who underwent an 8-week training course (weekly 1.5 hour sessions), followed by an evaluation [32].

#### Case identification and hearing assessment

In four studies, case identification occurred via community-based screening, sites for which were provided by local government and/or NGOs, and with portable equipment [28, 29, 33, 34]. In one study, hearing screening was conducted through hearing aids that produce pure tones for audiometric screening when connected to a smartphone application via Bluetooth. These hearing aids were covered by circumaural earmuffs to minimize the effect of environmental noise, and were the same hearing aids that were later used to fit the patients [34]. Other studies used portable computer- or tablet-based audiometers [30–32].

In two studies (part of the same randomized trial), participants either underwent a hearing assessment i) in the clinic, performed by a qualified hearing care provider in a sound-proof room, or ii) in a quiet place in their home, performed by a trained non-specialist [28, 29]. Most studies provided limited information on hearing assessment, as assessments were often only to determine study or hearing aid fitting eligibility and were not a primary focus of the research.

#### Hearing aid details

Varying levels of hearing technology were used. Two studies used pocket model analog hearing aids coupled to domes or custom earmolds [28, 29]. Three studies fitted participants with digital or semi-digital behind-the-ear hearing aids [30, 33, 34], and two of these studies specified that hearing aids were coupled to domes [30, 34]. In two studies, participants chose between low-cost, over-the-counter hearing device options [31, 32]. Five studies specified that devices were provided at no out-of-pocket cost to participants [30–34].

#### Hearing aid fitting

Five studies provided details on hearing aid fitting processes. Two studies specified hearing aids were fitted to NAL prescription targets [33, 34]. In one study, hearing aids were fitted via telehealth, in which the facilitator (trained non-specialist or qualified hearing care provider, depending on study arm) was with the participant and the qualified hearing care provider was in a remote clinic [30]. With the support of the qualified hearing care provider, trained non-specialist facilitators prepared and adjusted physical components of hearing aids (e.g., tubing, domes) and assisted with hearing aid programming and verification [30]. The qualified hearing care provider counseled participants on hearing aid use, communication, and expectation management, and the trained non-specialist facilitator provided counseling on hearing aid insertion/removal, features (e.g., volume control), and cleaning [30].

In two studies (pilot and follow-up randomized trial), participants underwent an intervention that aimed to enhance communication-related self-efficacy [31, 32]. Participants selected an over-the-counter hearing device, were fitted with and oriented to the device, and received education on age-related hearing loss and rehabilitation (e.g., communication strategies, expectation management) [31, 32].

#### Hearing aid follow-up and counseling

While all studies included some details on follow-up services, three focused only on study outcome data [28, 29, 32], whereas four, which are described next, focused on service delivery [30, 31, 33, 34]. Three studies provided telephone follow-up, either as needed [33], at scheduled intervals (8, 20, and 43 days after fitting) [34], or within 5 days of fitting [31]. In one study, participants additionally received text messages with information related to hearing aid use [34]. Across studies, in-person follow-up visits occurred at 2 weeks, 1, 3, and 6 months [33], 1.5 and 6 months [34], or 3 weeks [30] after hearing aid fitting. In-person visits were used to make minor adjustments to hearing aids [30, 33], complete interviews related to hearing aid use and outcomes [34] or provide additional counseling [30].

#### Outcomes

An overview of study outcomes is presented in S3 Table. Service delivery approaches were evaluated with researcher-developed questions, in terms of cost-effectiveness and health effects (measured by Disability-Adjusted Life Years [DALYs] averted) [29], and using standardized hearing aid outcome questionnaires (International Outcome Inventory for Hearing Aids [IOI-HA] [38], Abbreviated Profile of Hearing Aid Benefit [APHAB]) [28, 34, 39], hearing handicap (Hearing Handicap Inventory for the Elderly- Screening version [HHIE-S]) [31, 32, 40], and communication (Self-Efficacy for Situational Communication Management Questionnaire [SESMQ]) [30, 41].

Four studies reported the proportion of participants who used hearing aids regularly, defined >1 hour/day (59% and 75% of participants) [31, 32], >4 hours/day (80% of participants) [33], or daily use (88%) [34].

Two studies that were part of same trial compared community- and center-based service delivery, and reported similar hearing aid outcomes (IOI-HA scores) and DALYs averted for both approaches [28, 29]. However, the community-based model had less than half the costs of the center-based model [28, 29]. Two feasibility studies (without a control group) reported favorable hearing aid outcomes on the IOI-HA [34] and APHAB [33].

Two studies (pilot and follow-up randomized trial) reported a significant reduction of hearing handicap (HHIE-S) after participants underwent the hearing intervention [31, 32]. In the pilot study, participants who completed the intervention reported improved communication, social-emotional function, and depressive symptoms [31]. In the follow-up randomized trial, participants who underwent the intervention showed significant improvements on secondary outcomes of physical health-related quality of life, and listening self-efficacy, but not on those related to loneliness, depression, valuation of life, social isolation, or technology-related self-efficacy [32].

In another study, participants experienced improved communication self-efficacy (SESMQ) after hearing aid fitting, but there were no differences for the experimental (fitted by trained non-specialist CHWs) and control (fitted by qualified hearing care providers) arms [30]. Authors reported hearing aid delivery via telehealth and facilitated by on-site trained non-specialists was feasible and well-accepted by study participants [30].

### Telehealth

Four studies used telehealth for service delivery involving hearing aids in resource-limited settings (Table 3, S3 Table). Two case studies described hearing aid provision through telehealth [42, 43]. One pilot study described the development of a hybrid (combination of telehealth and in-person services) audiology clinic in South Africa [44], and a case-control study compared outcomes for patients who received hearing-related treatment in the clinic or via telehealth [45]. Two studies were conducted in LMIC [42, 44] whereas 2 were conducted in high-income countries in resource-limited settings in which most persons resided in rural settings [43, 45]. Three studies were conducted in adults [42, 44, 45], and one study did not specify patients’ ages [43].

**Table 3:** Details on service provision for studies using telehealth.

First Author (yr) Country (Income level)	Service provider Services provided directly to patient or through facilitator	Program development & training	Case identification & hearing assessment	Hearing aid details	Hearing aid fitting	Hearing aid follow up and counseling
Penteado (2012) Brazil (Upper-middle)	Remote and in clinic qualified hearing care providers (audiologists). Services provided directly to patient and through qualified hearing care provider facilitator.	Study team developed training protocols relevant to telehealth, which were used to train remote audiologist.	---	Patients’ hearing aids	*** 2 patients: Remote audiologist updated patient data, and fitting was performed by in-clinic audiologist. 1 patient: Remote audiologist performed fitting (supervised by in-clinic audiologist)	---
Pearce (2009) Australia (High)	Remote qualified hearing care provider (audiologist) and trained non-specialist facilitator. Services provided through trained non-specialist facilitator.	---	*** Trained non-specialist facilitator situated patient. Remote qualified hearing care provider conducted assessment using video otoscopy and remote audiometer.	Patients’ hearing aids	*** Trained non-specialist facilitator connected hearing aids and placed real-ear measurement probes. Remote qualified hearing care provider made changes to hearing aids, remotely.	*** Remote qualified hearing care provider counselled patient during appointment.
Ratanjee-Vanmali (2019) South Africa (Upper-middle)	Qualified hearing care provider (audiologist) Services provided directly to patient.	Development of virtual audiology clinic	Screened online with digits in noise test.	---	Fitting and verification conducted in face-to-face appointment.	*** Continuous face-to-face and online support offered by audiologist. An online aural rehabilitation program was offered.
Pross (2016) USA (High)	Remote qualified hearing care provider (audiologist) and trained non-specialist facilitator.	---	*** Trained non-specialist facilitator situated patient. Remote qualified hearing care provider conducted assessment (video otoscopy, audiometric threshold testing, otoacoustic emissions, immittance, and speech testing).	---	*** Trained non-specialist facilitator situated patient. Remote qualified hearing care provider conducted hearing aid fitting and verification.	---

*** Service was provided by telehealth.

The number of studies that provided telehealth services for each step of hearing aid service delivery is as follows: hearing evaluation, 2 [43, 45]; hearing aid fitting, 3 [42, 43, 45]; and hearing aid follow-up and counseling, 2 [43, 44].

#### Service provider

In one study, qualified hearing care providers provided services directly to the patient [44], and in three studies, in-person services were provided by a facilitator under the support of a remotely located qualified hearing care provider. Facilitators were trained non-specialists [43, 45], or qualified hearing care providers [42].

#### Program development and training

Two studies described program development and/or training of providers related to telehealth [42, 44]. More specifically, one study outlined the processes of establishing a hybrid audiology clinic that included telehealth and in-person services [44]. Another study detailed that a telehealth facilitator was trained online by a remotely located audiologist, and that the training focused on hearing aid features and fitting processes [42].

#### Case identification and hearing assessment

Two studies used telehealth for hearing assessment, both of which involved a remotely located qualified hearing care provider and an in-person trained non-specialist facilitator [43, 45]. The hearing assessment, which included video otoscopy and pure-tone audiometry, was conducted remotely by the qualified hearing care provider, and the non-specialist facilitator helped prepare the patient for testing [43, 45].

#### Hearing aid details

Two studies specified that participants used their own hearing aids [42, 43].

#### Hearing aid fitting

Three studies described hearing aid fitting via telehealth [42, 43, 45]. One case study in Brazil presented results of three hearing aid fitting sessions, each of which lasted approximately 15–20 minutes and were conducted by the remotely located qualified hearing care provider (2 cases) or the on-site facilitator (1 case) [42]. Another case study presented results of one fitting session conducted in remote Australia [43]. In this case, an in-person trained non-specialist facilitator connected the patient’s hearing aid to the computer and placed the real-ear measurement probe in the ear, and the remotely located qualified hearing care provider adjusted the hearing aids appropriately [43].

In the case-control study, a remotely located qualified hearing care provider programmed and adjusted patients’ hearing aids and conducted real ear measures [45]. The on-site trained non-specialist facilitator assisted the audiologist by, for example, placing real ear probe microphones in patients’ ears [45].

#### Hearing aid follow up and counseling

Two studies described hearing aid follow up and/or counseling in the context of telehealth. One study described two cases of telehealth audiology care that included hearing aid follow-up services [43]. In both cases, a remote qualified hearing care provider, supported by an in-person trained non-specialist facilitator, made fine-tuning adjustments to patients’ hearing aids or provided counseling. Authors noted that in one case, the availability of telehealth services reduced the wait time for a patient by two months [43]. In the study that described the development of a hybrid audiology clinic, on-demand online support was offered to patients directly by a qualified hearing care provider, and patients were recommended to complete an online aural rehabilitation program [44].

#### Outcomes

Details on study outcomes are in S3 Table. Three case or pilot studies indicated it was feasible to conduct virtual trainings for facilitators [42], provide hearing aid services to patients located in remote regions [43], and to use a mixed model of hearing aid service delivery that incorporated in-person and telehealth services [44]. Another study showed there were not substantial differences in hearing aid satisfaction (IOI-HA scores) for patients fitted with hearing aids in person or via telehealth, thus supporting the feasibility of telehealth services [45].

## Discussion

This systematic scoping review presents evidence on hearing aid service delivery approaches in LMIC and resource-limited settings. A thorough understanding of the evidence base supporting different approaches to service delivery could be used to inform decision-making related to hearing aid service delivery approaches in other LMIC and resource-limited settings. In the studies included in this review, hearing aid provision occurred in hospitals and clinics, through large-scale donation programs supported by philanthropic organizations, and in community-based settings, and in some cases, was supported with telehealth. Hearing aid services were provided by qualified hearing care providers, such as audiologists or ENT physicians, but also by trained non-specialists, such as CHWs and facilitators supporting telehealth services. Next, we synthesize findings for each service delivery approach, separately and together, to highlight key considerations for quality and sustainable hearing aid service delivery approaches.

### Hospital-based (central) service delivery and large-scale donation programs

Two studies in this review demonstrated how hospital-based audiology departments providing quality services can be successfully established and sustained in LMIC [23, 24]. However, another study showed that persons who received hearing aids from public hospitals experienced poor hearing aid outcomes, which may be attributable to poor access of hospital-based services, caused by factors such as limited transportation to the hospital and language barriers [25]. Consistent with those findings, past research has highlighted the barriers of relying on hospital-based care to provide hearing-related services to individuals in rural areas [46]. On a similar note, one study described limitations of large-scale hearing aid donation programs in a LMIC, including poor outcomes related to hearing aid fitting, quality, and maintenance. Authors highlighted that while such programs are efficient, they may offer a sub-optimal and short-term solution to managing hearing loss in LMIC [26].

Hospital-based services often rely on qualified hearing care providers [23–26]. Although there were examples of audiology departments successfully and sustainably training new qualified hearing care providers, and using a task sharing approach to train non-specialist providers [23, 24], importantly, there remains an inadequate number of professionals globally who can provide hearing services [2, 3, 47–49].

Hospital-based approaches to service delivery are valuable in certain settings and can facilitate specialized care. However, their effectiveness is limited in providing services in rural and remote areas, where approximately 50% of individuals in LMICs reside [50]. Therefore, hospital-based care could be a resource center to support non-specialists providing services in community-based or satellite facilities that utilize telehealth, to improve the reach of hearing aid services.

### Community-based service delivery

Across the studies in this review, it was feasible for trained non-specialists in community-based settings to provide services across the continuum of audiological care, including hearing assessment, earmold impressions, hearing aid fitting and adjustment, counseling and follow up, and hearing aid maintenance and minor repairs [28–34]. There were few studies that compared community- and clinical-based services. Importantly, as compared to clinical-based services, community-based services yielded similar outcomes related to hearing aid use and satisfaction, and DALYs averted [28–30] and were shown to be more cost-effective [29].

Community-based care facilitates the use of task sharing, which refers to the redistribution of clinical tasks or some of their components among different cadres of health workers [51, 52]. In hearing health care, tasks can be shared among qualified hearing care providers and trained non-specialist providers, to improve access to care while ensuring the provision of quality services. Community-based care is also supported by use of digital technologies, including mobile health, that can facilitate care provision by non-specialists [53–56]. For example, one study in this review demonstrated how portable mobile health technologies can support hearing assessment, hearing aid fitting, and follow-up [56]. Authors described how mobile health technologies supported community-based care, as trained non-specialist providers could travel to communities and provide in-home services with minimal, easy-to-use equipment that did not require extensive training to operate [56].

These concepts are in line with recommendations from the WHO World Report on Hearing, which recommends implementation of task sharing and innovations in hearing technologies to improve global hearing health care access [2]. While this review focused on hearing aid provision, other research has indicated trained non-specialists can provide additional hearing-related services in community settings, including those related to hearing loss prevention, hearing screening and assessment, and rehabilitation [19, 54, 57, 58].

### Telehealth

Providing hearing aid services via telehealth, including hearing assessment, hearing aid fitting, and follow-up and counseling, appears feasible in LMIC or resource-limited settings. Across studies, hearing aid services were delivered synchronously, except for an online aural rehabilitation program offered to patients of a hybrid audiology clinic [44]. In most studies, hearing aid services were delivered with assistance from a trained non-specialist facilitator, although in one study, audiologists provided services directly to patients [44]. Two studies in the ‘Community-based care’ section of this article also demonstrated feasibility or providing asynchronous (i.e., counseling support to mobile phones) and synchronous remote follow-up [30, 34].

Importantly, few studies focused on the use of telehealth to provide hearing aid services in LMIC or resource-limited settings, and most were case studies. Yet, the feasibility of providing hearing aid services via telehealth is reinforced by research conducted in high-income countries, which support the feasibility and efficiency of telehealth services, and show that those who receive telehealth services experience positive hearing aid outcomes on communication and quality of life [20, 59, 60].

### Synthesis

Key findings from this review are that hearing aid services can be successfully delivered in community-based settings and remotely, and that trained non-specialists can provide quality hearing aid services, which can help to overcome human resource limitations [2, 47–49]. Task sharing is a crucial strategy to overcome the global dearth of qualified hearing care providers, such as audiologists and ENT physicians, worldwide [2, 52, 61, 62]. Hearing aid provision is only one part of a comprehensive ear and hearing care program. Therefore, service delivery approaches related to hearing aids must include the components of testing, follow up and related services; be harmonized with ear and hearing care programs; be sustainable; and be optimized to reach the desired target population in a given setting.

Community-based and remote (telehealth) care can improve access to hearing aid services [63]. Hospital-based hearing health care can also provide quality audiological services to some individuals, and importantly, can serve as referral centers in cases where it is not appropriate to provide hearing aid-related services in the community. Studies in this review showed it was feasible to provide hearing aid service to both adults and children using the approaches described above. As mentioned above, service delivery approaches must be appropriately tailored to the target population. For example, programs including or focused on children must be sensitive to diagnosis of pathologies common in children, such as otitis media, and must ensure hearing aid fittings facilitate their regular use, in order to minimize the impacts hearing loss for children [7, 64].

There is a need for effective, low-cost, high-quality technology that supports hearing aid service delivery in LMIC or resource-limited settings by non-specialist providers [46, 53–56, 65]. Currently available technologies for use in community settings include portable equipment and innovative technologies supported by mobile health [53, 54, 56]. This can support service delivery by non-specialist providers in community-based settings because the equipment is easy to travel with and often does not require extensive training to operate. Low-cost and high-quality hearing aid technologies could also reduce the reliance on donated or used hearing aids. This can mitigate the ethical considerations tied to the use of donated hearing aids, such as limited access to (i) audiological follow up, (ii) hearing aid replacement, and (iii) batteries and accessories, as well as the fact that choices related to hearing aid selection and fitting are often dictated by availability of hearing aids [65].

Low-cost and pre-programmable hearing aids can also support community-based service delivery [54]. Nearly all studies in this review used behind-the-ear hearing aids, rather than those that require more customization, such as in-the-ear or in-the-canal types, likely because behind-the-ear hearing aids can be appropriately fit to many different patient profiles. Along these lines, pre-programmable hearing aids contain pre-set amplification protocols developed based on common configurations of hearing loss while still allowing for volume adjustment [66]. A recent report, using data from 23 sites across 16 LMIC, suggests that pre-programmable hearing aids have the potential to yield positive outcomes in LMIC, and suggests that it is feasible to incorporate pre-programmable hearing aids into service delivery approaches [67]. Importantly, the use of such technologies can optimize hearing aid fitting processes and allow for trained non-specialists to effectively provide quality services.

### Strengths and limitations

A strength of this study is that to our knowledge, this is the first article to comprehensively review service delivery approaches for hearing aid provision with a focus on LMIC and resource-limited settings. Another strength is that results from this study can be used to inform decision-making related to service delivery approaches in LMIC and resource-limited settings. Limitations of this review are as follows. Given the nature of this scoping review, we did not assess risk of bias for the included studies. While we conducted this review in English, French, and Spanish, there may be other relevant articles published in other languages that were not identified through our search.

This scoping review reflects the limitations of the studies included. Studies used various methods to determine the success or feasibility of service delivery approaches. Outcomes were reported at various follow-up times up to 6 months after hearing aid fitting, and long-term outcomes were not reported. Methodological differences make it challenging to compare results across studies and demonstrate the need for standardized methods in data collection and reporting. As stated above, most studies were case or feasibility studies, some were conducted in small samples of individuals, and most were conducted in samples of adults. Studies were conducted in relatively small geographic areas, although they included data from nine countries. These traits may limit generalizability of study findings. There were only two studies that directly compared community- versus center-based approaches [28, 29]. While they demonstrated the effectiveness of community-based approaches, it may not be possible to extrapolate results to other settings. Taken together, these limitations emphasize the need to tailor service delivery approaches to the population of interest, and furthermore, provide opportunities for future research to fill these gaps.

## Conclusions

Results from this systematic scoping review support the feasibility and effectiveness of hearing aid service delivery approaches that can improve access to hearing aids in LMIC and resource-limited settings. More specifically, studies supported the feasibility and effectiveness of community-based care, and the feasibility of telehealth, and leveraging trained non-specialist providers, by use of task sharing, to overcome limited human resources trained in ear and hearing care. These approaches, which should be supported by low-cost and quality innovative technologies and by the sustainable training of new providers, can help to improve access to hearing aid technologies, thereby reducing the global burden of hearing loss.

## Supporting information

S1 Text Search strategy by database. (DOCX)

S1 Table Details for studies conducted in hospital-based settings or large-scale donation programs. (DOCX)

S2 Table Details for studies conducted in community-based settings. (DOCX)

S3 Table Details for studies using telehealth. (DOCX)
