# Supplementary material for: Service delivery approaches related to hearing aids in low- and middle-income countries or resource-limited settings: A systematic scoping review
Source: PLOS Glob Public Health. 2024 Jan 24;4(1):e0002823. doi: 10.1371/journal.pgph.0002823 (PMC10807760; doi:10.1371/journal.pgph.0002823)
Supplement: S1 Text — (DOCX) [file pgph.0002823.s001.docx]

**S1 Text: Search strategy by database: June 2, 2022**

**1. PubMed**

Advanced search

n= 58

Filters: English, years 2000-2022

(“delivery of health care" OR “health delivery" OR “health care delivery” OR “healthcare delivery” OR “care delivery” OR "service provision" OR “service delivery” OR “telehealth” OR “teleaudiology” OR “telemedicine” OR “community health” OR “community-based” OR “hearing aid program*” OR “task sharing” OR “task shifting”)

AND (“hearing aid*" OR “assistive listening” OR “aural rehabilitation” OR “hearing device” OR “hearing instrument”) AND (“low and middle income countr*” OR “low to middle income countr*” OR “low income countr*” OR “middle income countr*” OR “low resource” OR “LMIC” OR “remote” OR “rural” OR “limited resource” OR “developing countr*” OR “Africa” OR “Asia” OR “Latin America”)

**2. Scopus**

Advanced search

n= 316

Filters: English, years 2000-2022

( ALL ( "delivery of health care" OR "health delivery" OR "health care delivery" OR "healthcare delivery" OR "care delivery" OR "service provision" OR "service delivery" OR "telehealth" OR "teleaudiology" OR "telemedicine" OR "community health" OR "community-based" OR "hearing aid program*" OR "task sharing" OR "task shifting" ) AND TITLE-ABS-KEY ( "hearing aid*" OR "assistive listening" OR "aural rehabilitation" OR "hearing device" OR "hearing instrument" ) AND ALL ( ( "low and middle income countr*" OR "low to middle income countr*" OR "low income countr*" OR "middle income countr*" OR "low resource" OR "LMIC" OR "remote" OR "rural" OR "limited resource" OR "developing countr*" OR "Africa" OR "Asia" OR "Latin America" ) ) )

**3. Ovid MEDLINE**

Advanced search

n= 54

Filters: English, years 2000-2022

((delivery of health care or health delivery or health care delivery or healthcare delivery or care delivery or service provision or service delivery or telehealth or teleaudiology or telemedicine or community health or community-based or hearing aid program* or task sharing or task shifting) and (hearing aid* or assistive listening or aural rehabilitation or hearing device or hearing instrument) and ((low and middle income countr*) or low to middle income countr* or low income countr* or middle income countr* or low resource or LMIC or remote or rural or limited resource or developing countr* or Africa or Asia or Latin America)).mp. [mp=title, abstract, original title, name of substance word, subject heading word, floating sub-heading word, keyword heading word, organism supplementary concept word, protocol supplementary concept word, rare disease supplementary concept word, unique identifier, synonyms]
