# Supplementary material for: Service delivery approaches related to hearing aids in low- and middle-income countries or resource-limited settings: A systematic scoping review
Source: PLOS Glob Public Health. 2024 Jan 24;4(1):e0002823. doi: 10.1371/journal.pgph.0002823 (PMC10807760; doi:10.1371/journal.pgph.0002823)
Supplement: S1 Table — (DOCX) [file pgph.0002823.s002.docx]

S1 Table: Details for studies conducted in hospital-based settings or large-scale donation programs

| **First Author (yr)**  **Country (Income level)** | **Purpose** | **Study design** | **n, age group** | **Details, service provision** | **Primary outcome definition related to service delivery** | **Primary outcomes or results** |
| --- | --- | --- | --- | --- | --- | --- |
| Carkeet (2014)  Dominican Republic (Upper-middle) | Present case study of EARs Inc., a program that developed audiology clinics in partnership with government and NGOs. | Case Study | n= n/a  2 clinics serve children & adults. 1 clinic serves only infants. | Services provided in the clinic, though some community outreach occurs. Hearing aids fitted by qualified hearing care providers. Patients attend 2 follow-up appointments. | n/a (case study) | Overview of the development and sustainment of a central audiology clinic. |
| Parmar (2021)  Malawi (Low) | Describe patient profile visiting clinic in Malawi.  Provide details on hearing aid provision at Queen Elizabeth Central Hospital audiology clinic. | Case Study  Retrospective record review | *n= 2299 patients assessed*  Adults: 18-94 yr | Services provided in hospital. NGO assisted in training qualified hearing care providers and trained non-specialists. | n/a (case study) | Overview of the development and sustainment of an audiology clinic embedded in tertiary hospital. |
| Sooful (2009)  South Africa (Upper-Middle) | Examine maintenance & utilization of hearing aids provided to patients in government hospitals.  Report factors that impacted the hearing aid service delivery. | Mixed methods | *n=57*  Age: n/a^1^ | Hearing aids provided at secondary or tertiary hospitals. | Questionnaire on maintenance, utilization, and condition of hearing aids.  Open-ended questions on hearing aid service delivery. | Maintenance: 26% reported ever having cleaned hearing aids.  Utilization: 12% daily use.  Condition: Many hearing aids needed repair or replacement.  Factors that impacted service delivery: finance, language barriers. |
| Newall (2019)  Philippines (Lower-middle) | Evaluate effectiveness of large-scale hearing aid donation programs, which used non-standard service delivery models to maximize efficiency. | Case study | *n=100*  Children (<18 yr) and Adults (≥18 yr) | Fitted with hearing aids during donation program organized by philanthropic organizations. Services provided by qualified hearing care provider. Patients fit in single day by going through stations: registration, ear screening, ear management, hearing aid fitting, batteries, checkout. | Proportion of patients fit within 5 dB of real ear target for thresholds: i) 0.5, 1.0, 2.0 kHz, and ii) 0.5, 1.0, 2.0, 4.0 kHz  Hearing aid management^2^  Performance measures | Real ear:  i) 2.7% fit to target, and ii) 0% fit to target.  40% reported difficulty with hearing aid management.  Performance measures:  89% able to change batteries.  83% able to switch aid on/off.  50% had no insertion issues.  83% able to alter volume. |

^1^Did not specify age; ^2^Defined by comfort in noise, mold comfort, feedback, difficulty with management, difficulty obtaining batteries, device use.

Abbreviations: NGO: Non-governmental organization; dB: decibel
