# Supplementary material for: Service delivery approaches related to hearing aids in low- and middle-income countries or resource-limited settings: A systematic scoping review
Source: PLOS Glob Public Health. 2024 Jan 24;4(1):e0002823. doi: 10.1371/journal.pgph.0002823 (PMC10807760; doi:10.1371/journal.pgph.0002823)
Supplement: S2 Table — (DOCX) [file pgph.0002823.s003.docx]

S2 Table: Details for studies conducted in community-based settings

| **First Author (year)**  **Country (Income level)** | **Purpose** | **Study design** | **n, age group** | **Details, service provision** | **Primary outcome definition related to service delivery** | **Primary outcomes or primary results** |
| --- | --- | --- | --- | --- | --- | --- |
| Borg (2018)  Bangladesh (Lower-middle) | Develop approach to provide hearing aids from trained non-specialists.  Compare effectiveness of community-based and center-based approaches for hearing aid fitting. | Cluster-randomized trial (ASHA trial) | *n=140*  Children: 12-16 yr | Community-based: trained non-specialists visit clients in their homes to fit hearing aids. Delivery similar to center-based except no custom earmolds provided. After 6 weeks, patient attended follow-up in clinic.  Center-based: client goes to hearing center and is seen by qualified hearing care provider. | Mean IOI-HA score of community-based vs center-based models | Similar scores (p>0.05) on 5/7 IOI-HA questions (use, improved activity, residual activity limitations, satisfaction, quality of life).  Center-based approach had higher mean IOI-HA scores (p<0.05) for questions on residual participation restrictions and impact on others. |
| Ekman (2017)  Bangladesh (Lower-middle) | Evaluate cost-effectiveness and effectiveness (in DALYs) of hearing aids provided by community- and center-based approaches. | Cluster-randomized trial (ASHA trial) | *n=142*  Children: 12-18 yr | Community-based: Trained non-specialists visit clients in their homes to fit hearing aids. Delivery similar to center-based except no custom earmolds provided. After 6 weeks, patient attended follow-up in clinic.  Center-based: client goes to hearing center and is seen by qualified hearing care provider. | Cost per treated patient   - Provider cost: staff, capital costs, equipment, facility costs, running costs. - Patient and caregiver: time costs, direct costs.   Health effects   - DALY estimates. Health effect of hearing aid use estimated as change in DALY corresponding to an improvement in hearing level by one category. | Cost per treated patient   - Center-based: BDT 13718 (US 171.50) - Community-based: BDT 6333 (US 79.18)   Health effects   - Center-based: 6.93 DALY averted. - Community-based: 7.91 DALY averted. |
| Emerson (2013)  India (Lower-middle) | Evaluate feasibility of using trained non-specialists to identify hearing loss and provide hearing aids and associated services. | Experimental pilot study | *n=111*  Children & Adults: 14-70 yr | Screening camps identify eligible participants, who were fit with hearing aids by trained non-specialists. Hearing assessment was conducted in the field.  Few details provided on hearing aid provision. | APHAB  Hearing aid use | Significant improvements on communication subscales of APHAB.  80% used hearing aids regularly (>4 hrs/day). |
| Frisby (2022)  South Africa (Upper-Middle) | Evaluate feasibility of community-based model to provide hearing aids to adults in low-income communities using trained non-specialist CHWs and supported by mHealth technologies. | Feasibility | *n=40 fit with hearing aids*  Adults: > 18 yr | Hearing assessment, hearing aid fitting and post-fitting support provided by trained non-specialist CHW. Service delivery used technologies that allowed for mobile evaluation/ fitting via Bluetooth and follow-up and counseling with mHealth technologies. | IOI-HA  Feasibility | High scores on IOI-HA (no control group).  Trained non-specialist CHW successfully led all hearing assessment, referrals, hearing aid fitting, and post-fitting support. |
| Nieman (2017)  USA (High) | Evaluate a community-based intervention (HEARS), which includes provision of an OTC listening device using a protocol developed for trained non-specialist CHW. | Randomized control pilot study  Immediate vs delayed treatment groups | *n=15*  treatment group: n=8;  delayed treatment group: n=7  Adults: ≥ 60 yr | Services provided by trained interventionist (used protocol developed for trained non-specialist CHW). Services included selection of listening device (PSAP or pocket talker), fitting and orientation, education on age-related hearing loss, and aural rehabilitation. | Change in HHIE-S score from baseline to 3-month follow up | Median hearing handicap decreased for immediate (19 to 10 points) and delayed treatment (20 to 16 points) groups. |
| Nieman (2022)  USA (High) | Assess efficacy of HEARS intervention. | Randomized controlled trial  Immediate vs delayed treatment groups | n=151  treatment group: n=78;  wait-list control group: n=73  Adults: ≥60 yr | Same as Nieman (2017) | Change in HHIE-S score from baseline to 3 months post-randomization. | HHIE-S scores significantly improved in the intervention (baseline: 21.7, follow up: 7.9 points) vs control (baseline: 20.1, follow up: 21 points) group. |
| Coco (2022)  USA (High) | Evaluate feasibility of trained non-specialist CHW as patient-site facilitators to provide hearing aid services via telehealth. | Randomized controlled trial | n=28  Experimental: n=14;  Control: n=14  Age: range 55 - 89 yrs (mean 73.9 yr) | Experimental: received telehealth hearing aid services with patient-site support from trained non-specialist CHW.  Control: received telehealth hearing aid services with patient-site support from trained qualified hearing care providers.  2 intervention visits – hearing aid fitting and follow up, 2 other outcomes over 17 weeks. | SESMQ | Both experimental and control groups showed significantly improved SESMQ scores from baseline to 17-week follow-up (p<0.01). There was no observed difference between groups. |

Abbreviations: CHW: Community health worker; OTC: over the counter; DALY: Disability-adjusted life year; IOI-HA: International Outcome Inventory for Hearing Aids; PSAP: Personal sound amplification device HHIE-S: Hearing Handicap Inventory for the Elderly- Screening version; SESMQ: Self Efficacy for Situational Communication Management Questionnaire; APHAB: Abbreviated Profile of Hearing Aid Benefit
