# Supplementary material for: Service delivery approaches related to hearing aids in low- and middle-income countries or resource-limited settings: A systematic scoping review
Source: PLOS Glob Public Health. 2024 Jan 24;4(1):e0002823. doi: 10.1371/journal.pgph.0002823 (PMC10807760; doi:10.1371/journal.pgph.0002823)
Supplement: S3 Table — (DOCX) [file pgph.0002823.s004.docx]

S3 Table: Details for studies using telehealth

| **First Author (year)**  **Country (Income level)** | **Purpose** | **Study design** | **n, age group** | **Details, service provision** | **Primary outcome definition related to service delivery** | **Primary outcomes or primary results** |
| --- | --- | --- | --- | --- | --- | --- |
| Penteado (2012)  Brazil (Upper-middle) | Evaluate benefits and limitations of tele-audiology for hearing aid fittings. | Case study | *n=3*  Adults: 61-81 yr | Patients fitted in person by a remotely located qualified hearing care provider (audiologist) supervised by an audiologist located in a clinic. | Feasibility | Feasible to fit hearing aids using telehealth, and to conduct virtual trainings for audiologists in different settings. |
| Pearce (2009)  Australia (High) | Determine feasibility of hearing assessment and hearing aid fitting to patients in remote areas using telehealth. | Case pilot study | *n=5*  Age n/a | Qualified hearing care provider (audiologist) based in a clinic in a main city center provided services to clients in remote communities with help of a trained non-specialist facilitator located in that community. | Feasibility | Feasible to conduct hearing assessment, hearing aid fitting, hearing aid adjustments, and rehabilitative counseling via telehealth. |
| Ratanjee-Vanmali (2019)  South Africa (Upper-middle) | Secondary objective was to describe set up and processes of virtual clinic. | Pilot | *n=51 (for hearing aid assessment)*  Adults: > 18 yr | Mixed model.   Most interactions were online. There were 2 face-to-face interactions in the patient’s home or office, or a satellite clinical site. | Feasibility | Mixed service delivery model (in person and online) was successful for hearing assessment, counseling, and support. |
| Pross (2016)  USA (High) | Assess hearing aid services (related to fitting and follow-up) provided in-person vs via telehealth. | Retrospective case-control | *n=42,679*  Adults: (Veterans) | Qualified hearing care provider located in clinic. Services provided remotely with help of trained non-specialist provider facilitator. | IOI-HA | Supports feasibility of providing hearing aid services (related to fitting and follow-up) via telehealth.  No substantial differences on hearing aid satisfaction (IOI-HA) between those fitted in-person or via telehealth. |

Abbreviations: IOI-HA: International Outcome Inventory for Hearing Aids
